# Climatic and Other Global Changes as Current Challenges in Improving Water Systems Management: Lessons from the Case of Italy

**DOI:** 10.1007/s11269-023-03424-0

**Published:** 2023-01-31

**Authors:** Giuseppe Rossi, David J. Peres

**Affiliations:** Department of Civil Engineering and Architecture, University of Catania, Via S. Sofia, 64, 95123 Catania, Italy

**Keywords:** Water resources, Drought risk, Flood risk, Water-energy-food nexus, Water legislation, Climate change

## Abstract

Climate change linked to human activities is affecting natural systems, increasing frequency and severity of water-related hazards. The issue of climate change pushes to tackle the expected risks in the water sector through a comprehensive and global view, with a revision of the paradigms considered as drivers of water resources development. Based on the evolution of these paradigms in Italy the main characteristics of an adaptive approach to climate change and other global changes are discussed relatively to water infrastructures, legislative and institutional frameworks. The objective of adaptation strategies is the increase of resilience of water systems, emphasizing the capability of reducing both physical and socio-political vulnerability, improving the governance of water services. Starting from the analysis of the evolution of water management, priorities for coping with future challenges are discussed with reference to the reduction of the risk of water shortage due to drought, to the mitigation of flood risk, and to the issues concerning the water-food-energy nexus.

## Introduction

The solution of water problems has been always recognized as a key issue to ensure the fundamental human right to water access and to achieve socio-economic development and environmental protection (Falkenmark 2001; Allan 2003; Rossi and Benedini 2020). The new concepts of sustainability and resilience emphasize the necessity of adopting adequate measures to face the global changes affecting water resources, such as climate change, land use modifications, and new economic, institutional and cultural features of society (Loucks 2022). In the past decades the idea of an “integrated water resources management” (IWRM), aimed at coping with multi-sources, multipurpose and complex decision making process, has been applied especially in water resources planning, by considering both technical solutions and economic, environmental and social aspects of water management (GWP 2000; Biswas 2004; UNEP 2012; Giakoumis and Voulvoulis 2018; Ibisch et al. 2016; Walker et al. 2015). However, the awareness of the increasing impacts of global changes on water systems has recently pushed on the need of shifting the current operation principles toward a more flexible and adaptive strategy, particularly in order to face the uncertainty and risk of extreme events and to improve communities’ preparedness (Garrote 2017). Such approach is confirmed by the lessons drawn from the Coronavirus pandemic, which has corroborated the need to face the uncertainty of the future, by improving the resilience of the society as the key-element of a comprehensive strategy for mankind (Antwi et al. 2021). In this review, we illustrate the evolution of the main paradigms that have guided the development of water resources in a country by applying specific principles of the sector as well as general principles derived from legislative, institutional and cultural framework at national and international levels, and how they can help to identify the strategies for an adaptive approach.

The paper is organized as follows. First, the evolution of paradigms at various stages of water resources development are described, with reference to the specific features of the case of Italy. In Section 3 we illustrate the concepts of adaptive management as a strategy to deal with global changes, as well as the paradigms for coping with water related disasters (drought and flood) and the water-energy-food nexus. Finally, in Section 4 concluding remarks for improving water system management in a context of global changes are drawn.

## Evolution of Paradigms at Various Stages of Water Resource Development

### Progress of water systems management

In its broadest sense, water resource management is defined as a comprehensive process aiming at achieving water resources use, water quality protection and defense against hydrological extremes. It includes the activities required for the development of water infrastructures (planning, design, construction, operation, maintenance, and control of structural facilities), as well as many non-structural instruments, such as the legislative framework and the institution's asset in decision making, including stakeholder and citizen participation.

Since 1970s', the notion of system has been introduced to analyze complex water problems; a system approach appeared a key tool to face the planning and operation of water resources (Hall and Dracup 1970; Texas Water Development Board 1971; Labadie 2004). A system can be defined as a collection of multiple elements that share a common goal and are linked mutually and tightly by a variety of inputs and outputs, including some type of feedback, with links to the external environment.

**Fig. 1:**
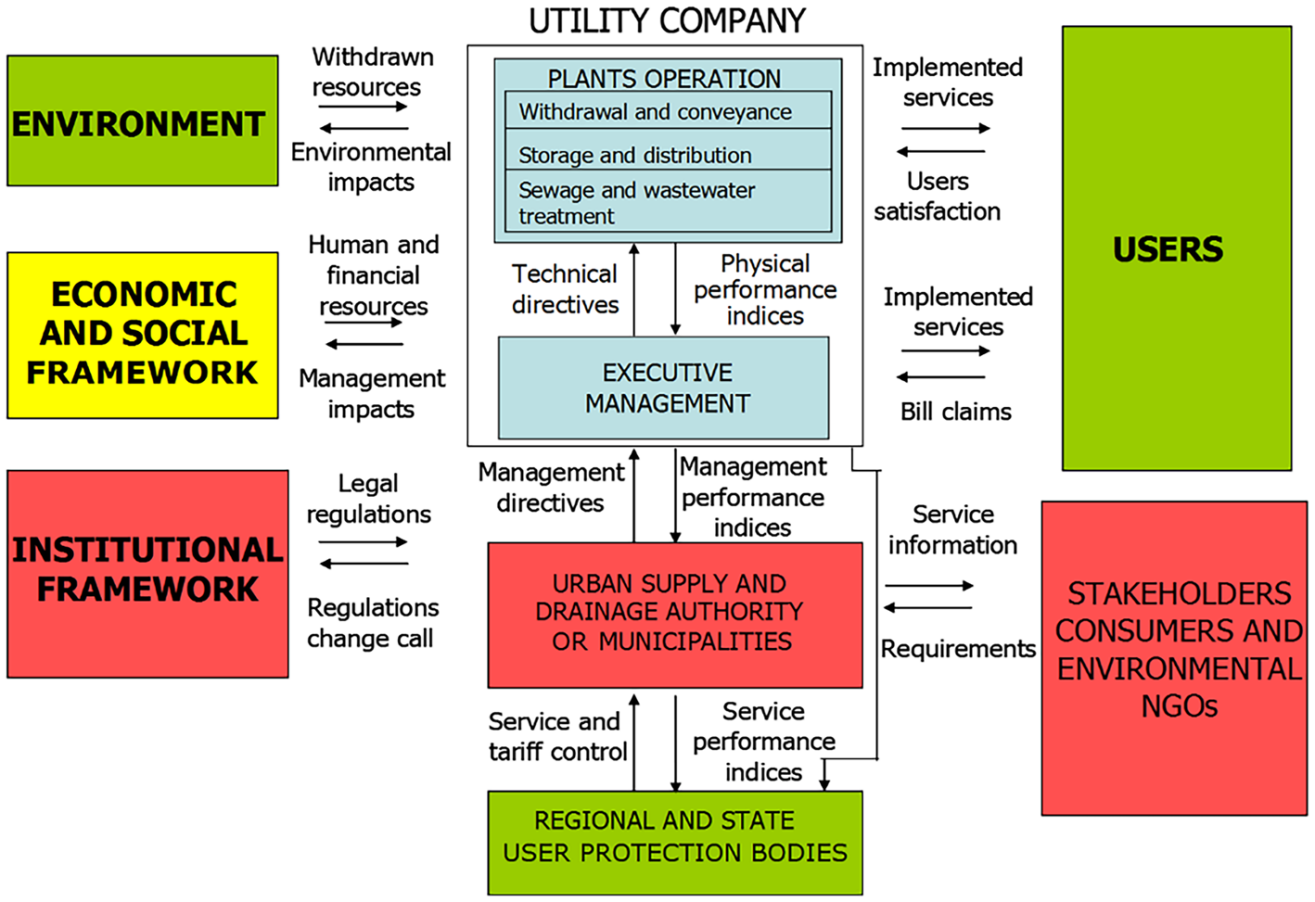
Interactions among an urban water system and external elements (adapted from Rossi and Benedini 2020)

Structural facilities of a water system consist of facilities that enable to match available resources, water needs (off-stream uses), and those that allow in-stream uses, while non-structural elements include regulation of water rights (permit systems), and legislative and institutional asset for governance of water sector, including the defense from flooding and landslides. It should be noted that in this way the concept of water system is broader than the physical components (natural water cycle and structural facilities). There are many complex multipurpose water systems in river catchments and urban environment. For instance, Fig. 1 presents a general scheme of an urban water system highlighting the complex interactions among the bodies which operate facilities, manage and govern services, and protect users’ rights, as well as the external elements (environmental, economic and legislative framework, users and other stakeholders).

In spite of the differences between the general frameworks of different countries, several common historical stages can be identified in the development of water resources systems, also due to the changes of general paradigms affecting culture and society.

Various interpretations have been given to the change in paradigms affecting the solution of water problems in the past. A simplified analysis, for example, distinguishes between a *water supply* model aimed at expanding resources devoted to various uses and a *water demand* management model aimed at decreasing water demands and/or developing recycling and reuse of wasted water in a circular manner.

Allan (2003) argued about the existence of three historical stages: i) *Pre-modernity*, connected to limited technical and organizational capacity; ii) *Industrial modernity*, characterized by engineering development in hydraulic works and large public or private investments (“hydraulic mission”), affecting in mid twentieth century both western liberal economics (Europe, USA) and planned economics of communists countries and then exported in developing countries; and iii) *Reflexive modernity*, inspired by concepts of environmental awareness, effort towards equity and public participation to the decision-making process.

Focusing on the experience in Italy, in the last one hundred and fifty years, three major stages of the evolution of water system management can be identified (Table 1).

**Table 1:** Stages of evolution of water resources development in Italy (From: Rossi and Benedini 2020)

**Stage/Main paradigm**		**View**	**Planning focus**	**Decision making features**
I	WR exploitation for human needs and economic growth	River basin as a hydrological and geo-morphological system	Structural measures to maximize yield to human needs and economic development of the country	• Large funding program by public • Top-down approach
II	Focus on WR quality and defense from water–related disasters	River basin as an ecosystem	Actions to reduce pollution and deterioration of natural rivers (with resistance against large structures as dams) and to mitigate flooding risk	• Concern on water quality and ecological requirements • Concern on flood disasters in river and urban areas • Bottom-up approach
III	Fostering an integrated sustainable and equitable WR management	River basin as a complex system (including social aspects)	Conjunct management of water and land by improving existing supply systems to maximize welfare in a social equitable way without compromising sustainability of ecosystems	• Effort to shift from fragmentation to coordination with stakeholders’ and general public participation • Meeting top-down and bottom-up approaches

In the following section, these three main stages will be discussed through a short description of some main water infrastructures, legislative frameworks, institutional assets of water services, which can be considered basic elements of political strategies of Italy.

### Water systems management stages in Italy

#### Water resources exploitation for human needs and economic growth

This phase was aimed at the exploitation of water resources in order to satisfy human needs and enhance the economic growth of the country. Here, the engineering and economic approach can be considered the prevalent driver not only of the infrastructures built in the country but also of the legislative acts and institutional setting. The development of water infrastructures has been matched by legislative framework evolution, with a shift of water resource management and soil conservation from a dominant private initiative to an expanding role of public administration in facility development and building. For instance, the Royal Decree 1775/1933 provided a regulation of private withdrawals for various uses through a “licensing rule”, which fostered for many years the growth of hydro-electric power and the extension of irrigation. Afterwards, a few Acts indicated the central government's incentive to increase the role of the State through more incisive planning activity. Examples in this direction are Law 184/1952, which established the first river regulation plan, and Law 129/1963 that identified the criteria for a drinking water master-plan.

#### Focus on water resources quality and defense from water-related disasters

This took place in a second phase of water systems development. In particular, Law 319/1976 ("Merli Act") regulated water pollution control, introducing standard quality levels for effluents and a sewerage and wastewater treatment charge. Also, regulations for "minimum instream flow" were introduced. The establishment of Law 319/1976 fostered the programs to construct sewerages and new wastewater treatment plants. Several initiatives against water-related disasters, particularly defense from floods and landslides and mitigation of drought risk, were launched, for instance with Law 183/1989. The National Group for Defense from Hydrogeological Disasters (GNDCI) activities, financially supported by the National Research Council, involving Italian universities and public research institutions, contributed to studies on flood mitigation and landslides, as well as the development of methodologies for estimating design-floods. Unfortunately, insufficient financial resources dedicated to flood prevention did not result in a significant reduction of flooding risk in many Italian regions. Most of investments for flood defense were funded by the Civil Protection (established by Law. 225/1992) to support emergency actions for rescue, assistance, recovery, temporary or permanent relocation and restoration of infrastructures and buildings.

#### Fostering integrated sustainable and equitable water resources management

In this phase, sustainability (Brundtland 1987) and ethical issues (Selborne 2000; Rossi 2015) were taken into account. For instance, the principle of “water access as human right”, recognized by the Assembly of United Nations in 2010, contributed likely to the success of the referendum on June 2011 against the privatization of municipal services. Also, an enhanced role of citizens and stakeholders in all decision-making processes has been invoked, starting from transparency in public administration acts to participation procedures. These principles were recognized by Law 36/1994 ("Galli Act"). Also, Law 267/1998 (Sarno Act) and Law 365/2000 (Soverato Act) tried to improve the measures for coping with floods and landslides, by means of Plans within the wider River Basin Plans introduced by Law 183/1989. In terms of pollution control, the Legislative Decree 152/1999 changed the earlier regulations of the Law 319/1976, introducing Regional Water Protection Plans. Finally, Legislative Decree 152/2006 maintained the earlier acts, with a few adjustments and improvements in accordance with contemporary European regulations. Environmental Impact Assessments were introduced by European Directive 85/337/EC and recognized by Italian law as necessary for the most important water works. Customer satisfaction was also taken into account by Law 290/1999, by means of a Service Chart for water supply consumers, updated in 2015 by the Authority for Electric Energy Gas and Water System (AEEGSI), now the Energy, Networks and Environment Regulation Authority (ARERA). Unfortunately, significant weaknesses of this development are present. These include: limited financial resources in the water field, complexity of the legislation and decision-making process with overlapping institutions, delays in the planning process and in the realization of water works, and a decreasing role of experts from universities and research institutions in assisting political decisions. Many ethical principles are reflected in legislation, even though there are some difficulties in their practical implementation.

## Global Changes and Adaptive Management

### Strategies of Adaptation to Climate Change and Global Changes

The limitations of current water management stood out clearly in the recent years with the increasing awareness of water managers, stakeholders and society about the necessity of responding to the challenges posed by climate change and other global changes, which require to increase the adaptive capacity to develop generalized responses. Thus, the transformation of the territory and the modifications in driving economic and social processes require to be analyzed in a broader perspective, in order to improve water management. Of course, the weaknesses of the water infrastructures – from the lack of maintenance of water works to an inadequate governance of water service – still plays an important role, but the solution cannot be limited to these aspects. Especially the positive strengths, – such as the technological innovations and digital advancements, as well as the new awareness about public participation to decision making process –, have to be considered in order to achieve a sustainable development with a real environment protection, and an equitable use of water. Adaptive water management is also invoked to deal with the water-energy-food nexus by linking the different strategies of development of each field. In particular, this can be achieved by reducing energy footprint of water facilities and water footprint of power generation systems, as well as by comparing the advantages and disadvantages of land assignation for food production or energy increase.

In this broad perspective, an *adaptive management* - “the ability to change management practices based on new experience and insight” (Pahl-Wostl 2007) – is crucial to improve the resilience of the water resource system in its physical, biological and human components. According to the same author, the term *adaptive water resource management* emphasizes the capability of a water system to reduce its physical and social vulnerability improving its “capability to adjust, via changes in its characteristics or behavior, so as to cope better with existing and future stress”. In other words, adaptive management is based on a pro-active approach aimed to increase the ability of the system to respond to change rather than reacting to undesirable impacts of change. This relates to the concept of *resilience* which is defined as “the ability of a system and its component parts to anticipate, absorb, accommodate, or recover from the effects of a hazardous event in a timely and efficient manner” (IPCC 2012).

However, with reference to the legislative-institutional framework at European level, several elements of weakness exist both in the legislation framework and in the procedures regulating decisional processes to cope with the challenges of global changes (Baranyai 2020). First, the statements of principle about the will of improving the resilience to climate change, present a lack of enforcement. This is the case, for instance, of the statement about the main objectives of “preventing and solving the conflicts, contributing to an equitable sustainable and integrated management of water resources”, by the Council of European Union. In fact, the European Directives in force aim at fighting water pollution and improving water quality in water bodies (Water Framework Directive 2000/60) and at mitigating flood risk (Floods Directive 2007/60). However, they don’t tackle some of the foreseen impacts of climate changes in Europe, such as the shortage risk due to drought and in general terms the increasing variability of streamflows, which will produce critical effects on the sharing of resources between the trans-frontier countries. A tool like the Albufeira Convention, which gives guideline to share water resources between Spain and Portugal in the case of high water shortages in the common basins, would be a good example to follow (Hervás-Gámez and Delgado-Ramos 2019). Another more significant point of weakness is related to the fact that the legal framework of EU does not facilitate the solution of controversies, as the Court of Justice precludes the recourse to the arbitration and to the International Court of Justice for water conflicts.

### Priorities for Mitigating Risk of Water Shortage Due to Drought

Drought is defined as a temporary condition of a severe reduction of water availability, lasting a significant amount of time and affecting a large region. Drought is a natural phenomenon, but it can be considered a disaster with severity of impacts depending on the vulnerability of the water systems, including infrastructures, preparedness to implement appropriate mitigations measures and economic and social aspects (Mishra and Singh 2010).

Drought risk has been traditionally managed by a *crisis management* approach. Since this approach is based on last-minute decisions, it generally leads to expensive actions, with unbearable environmental and social impacts. Thus, a shift towards a *risk management* approach, based on measures planned in advance, has been progressively advocated (Yevjevich et al. 1983; Wilhite 1987; Rossi 2017). Starting from the end of 1990s, such a shift has been emphasized in policy instruments adopted in drought-prone countries such as Australia (Botterill and Wilhite 2005), South Africa and USA (Wilhite et al. 2005). It is also suggested by International or European recommendations (UNISDR 2007; EC 2007, 2011), as well as promoted by research projects on drought (see, e.g., Medroplan 2007).

Another aspect is that drought management presents some differences with respect to managing other natural disasters: *i*) prevention actions may be effectively planned since drought effects evolve slowly along a large time span; *ii*) strategic measures for improving drought preparedness are generally more complex, since the spectrum of potential long-term actions is very large; and *iii*) operational measures, to be implemented at drought inception, require an adaptive response, due to the dynamic feature of phenomenon. In particular, the operational measures should take into account the uncertainty in drought evolution, which can yield a duration and a severity different from those considered in the planning stage (Rossi 2017), taking also into account the impacts of climate change (Simonovic 2017; Peres et al. 2019, 2020; Senatore et al. 2022).

Assessment of water shortage risk due to drought can be carried out with reference to two distinct objectives, namely: 1) reducing the vulnerability of the system within the strategic planning stage through appropriate prevention strategies; 2) improving the performance of water supply during droughts within the operation stage. When dealing with strategic planning, risk assessment should be “*unconditional”*, i.e., not referred to a particular state or condition of the system, and aimed at selecting the best long-term measures (Bonaccorso et al. 2007). In the case of the operation of water supply systems under drought conditions, the risk assessment has to be “*conditional*”, i.e., taking into account the current state/condition of the system. In this case the main problem is defining the status of the system with respect to predefined operational levels (e.g. normal, alert, alarm) in order to decide *when* and *how* to activate predefined sets of mitigation measures (e.g., rationing policies and/or use of additional water resources) able to prevent future severe shortages (Medroplan 2007).

In the stage of operating policy of a single reservoir or complex water supply system, the assessment of water shortage requires to consider the role of early warning systems and forecasting tools, which can be developed exploiting the links between precipitation and large-scale circulation patterns (Bonaccorso et al. 2015).

The most recent United Nations International Strategy for Disaster Reduction (UNISDR) recommendations, which have been included in the Sendai Framework for Disaster Risk 2015–2030, emphasize the primary responsibility of States to prevent and reduce disaster risk. Some legislation make reference to the Integrated Water Resources Management (IWRM) model, which was introduced during the United Nations Conference on Water in Mar del Plata (1977). Until recently, European water policy gave little attention to drought challenges in terms of technological and financial tools, as well as legal acts (see Water Framework Directive 2000/60). Only on 2007 the EU Water Scarcity and Drought Expert Network developed a guidance document on drought preparedness and mitigation proposing the adoption of “Drought Management Plans” (DMPs), within River Basin Management Plans (EC 2007; Rossi 2009).

The expected revision of the Water Framework Directive (WFD) and of the water scarcity and droughts strategy could include options to increase drought resilience. Also, the last updates of River Basin Management Plans should include the effects of climate change within basin planning. According to the White Paper on Adapting to Climate Change (CEC 2009), one of the strategies proposed to increase resilience to climate change consists in the improvement of the management of water resources.

**Fig. 2:**
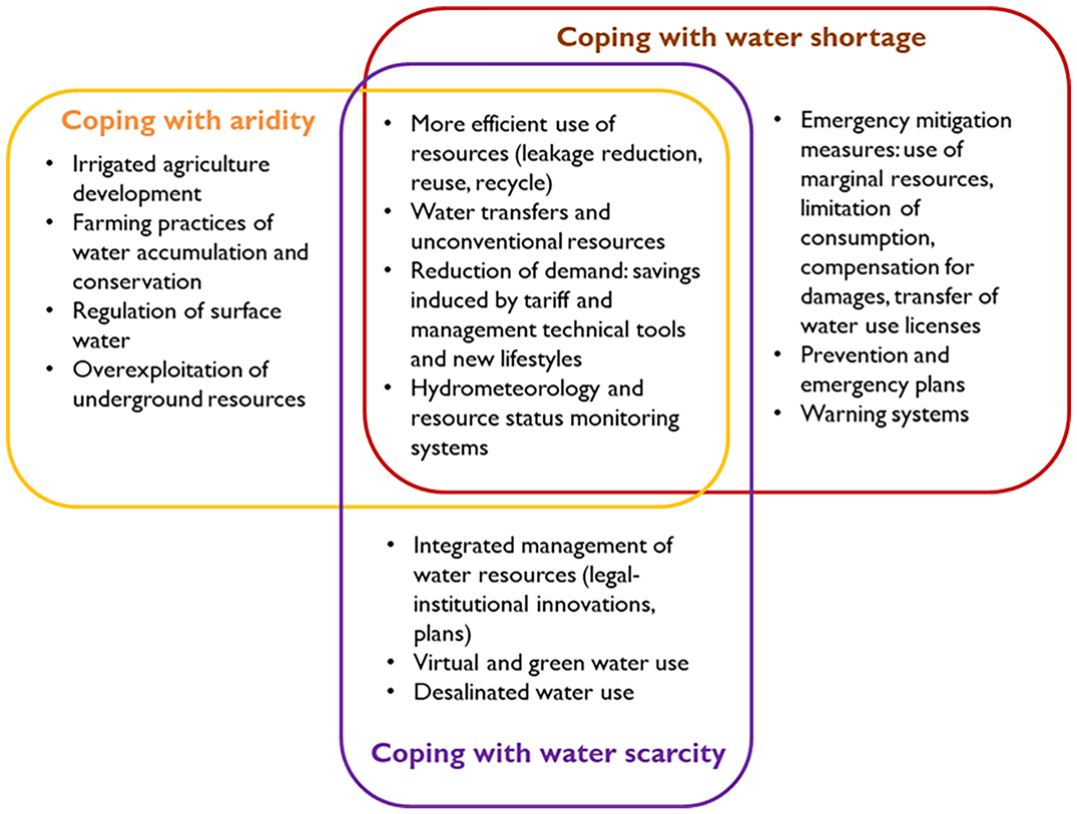
Measures for coping with water shortage due to drought and links with measures for coping with aridity and permanent water scarcity

More generally, measures for mitigation of shortage due to drought can be distinguished in three main categories, oriented at: *i*) increasing water supply, *ii*) reducing water demands and *iii*) minimizing drought impacts (Rossi 2017). They include *long term measures* and *short term measures*. In Fig. 2 some mitigation measures are shown, with their links to the strategies for coping with aridity and water scarcity.

Multi-criteria approaches have been proposed for selecting the best combination of measures, since the early 1980s (Duckstein 1983) and discussed or applied in several cases (see Munda et al. 1998, Rossi et al. 2005; Yilmaz and Harmancioglu 2010; Abdullah et al. 2021; Ihinegbu and Ogunwumi 2022).

Some lessons for a pro-active approach to cope with increase of drought risk due to global changes can be drawn from some recent experiences carried out in Italy, especially within the Po River basin (see Rossi and Benedini 2020), where a severe water crisis was faced by establishing a Technical Committee to manage water resources with real time monitoring and the assessment of drought evolution scenarios. The publication in July 2015 of a Water Balance Plan, contributed to reinforce the cooperation among the several institutions responsible for water resources management and the main stakeholders, as well as to minimize the impacts of water shortages. Key elements of this Plan were the assessment of available water reserves during spring and summer -- in order to optimize the allocation across different sources and water uses and reduce shortage risk during months with higher demands -- and a multiple stage early warning system.

From the Italian experiences, priorities for an effective approach to drought risk mitigation are the establishment of an appropriate institutional framework, a better coordination of national and local emergency management organizations and the development of advanced early warning systems. From the research standpoint, the following aspects should be addressed (Rossi 2017): a) better modeling of drought occurrence and characteristics; b) a thorough analysis of past experiences in drought monitoring and mitigation; c) advanced assessment of economic, environmental and societal impacts of droughts and mitigation measures, preferably based on multi-criterion tools; d) development of advanced tools for an “adaptive” drought management in water supply systems (drought early warning systems, drought monitoring and forecasting systems, and advanced DSS).

### Priorities for Mitigating Flood Risk

Mitigation of flooding risk can be considered one of the most relevant challenges to be faced by a community in order to improve its resilience to water-related disasters.

In Italy, the specific characteristic of a combined flooding and landslide risk explains the adoption of a more comprehensive view in Italian legislation in order to face both risks. Such an approach has been the basis of Law 183/1989 and subsequent regulations. While the establishment of the European Flood Directive 2007/60/EC, is focused only on flood risk, the activities aimed at understanding and managing the risks in Italy continue to concern both flood and landslide phenomena. This is a positive feature of the Italian legislative framework, which in the last decades has been characterized by a closer attention to non-structural measures and to the role of a civil protection organization. Furthermore, delays in the preparation and implementation of planning tools, limited financial resources devoted to structural interventions and cumbersome bureaucracy have influenced negatively the mitigation flood risk policies based on structural measures. The application of non-structural measures has also presented several difficulties, deriving from: *i*) a lack of coordination by flooding risk plans and those on land use; *ii*) a confusing decision-making process with separate responsibilities of civil protection and local authorities (mayor of a municipality); *iii*) limited public participation in the decision-making process ranging from strategy planning to the design of structural measures and to the operation of warning system.

A recent analysis of the measures for reducing flood risk in Italy (Rossi and Benedini 2020) has distinguished the categories of floods in: *i*) River flooding; *ii*) Urban flooding due to flash flood; *iii*) Flooding originating from dam and/or landslide; *iv*) Flooding connected with debris-flow or mud-slide. It has been ascertained that climate change is a factor in increasing flood risk, due to an increase in the frequency and the intensity of storms. Nevertheless, other different factors contribute to the growth of damages and causalities, such as: the increase of runoff due to the growth of impervious areas of catchments, the abandonment of agricultural practices, and the inadequacy of structural defense measures and river regulation infrastructures.

**Fig. 3:**
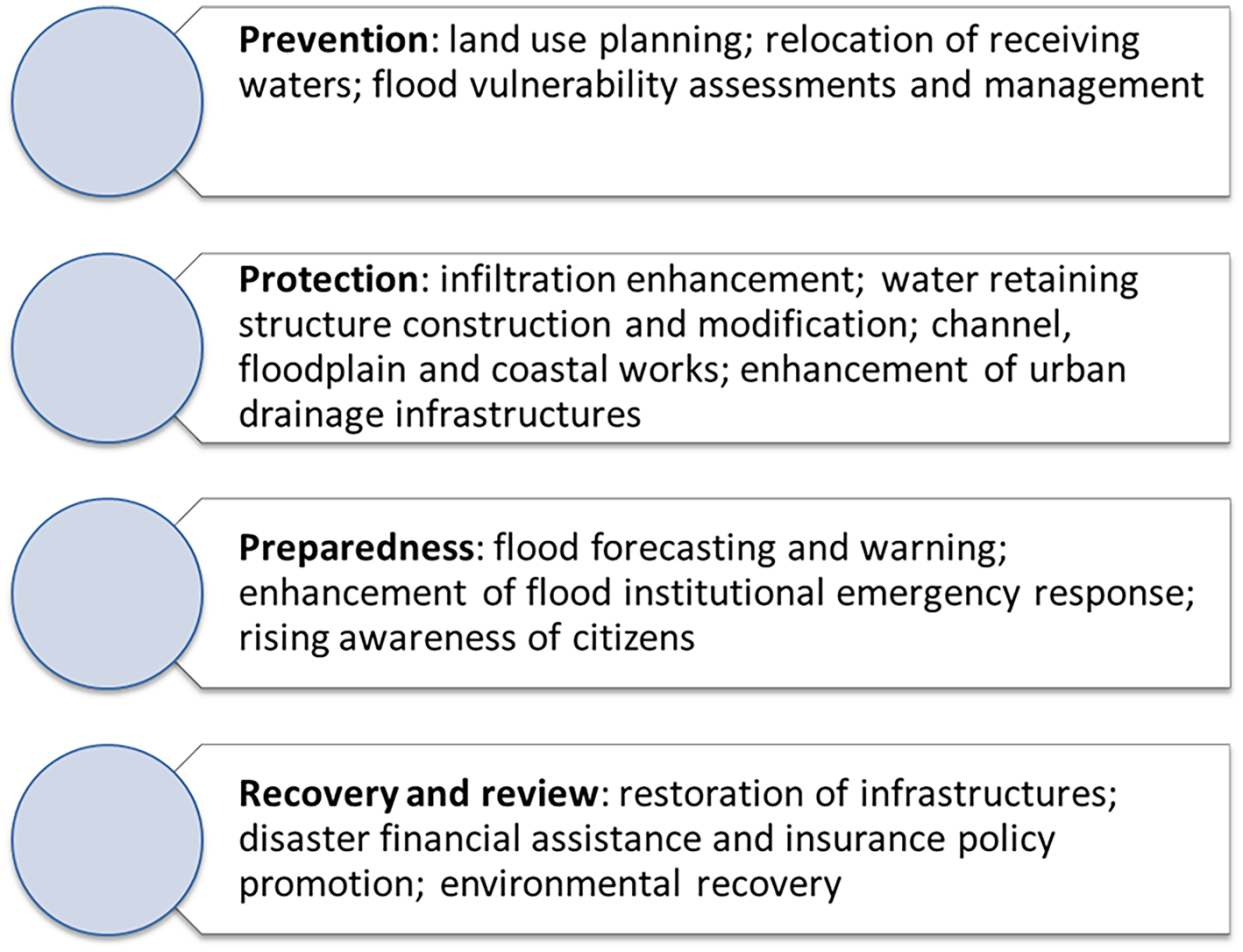
Adaptive management stages for flood risk mitigation

There is also an inadequacy of policy choices in the few last decades, which has not improved significantly the resilience of several urban areas and most of the basins to the flooding hazard. Finally, the inadequate behavior of people during the severe hydrological events is another significant reason of many casualties. Referring to the “Guidance for reporting under the Floods Directive” (EC 2013), flood management measures can be conveniently framed into the adaptive management stages presented in Fig. 3.

**Fig. 4:**
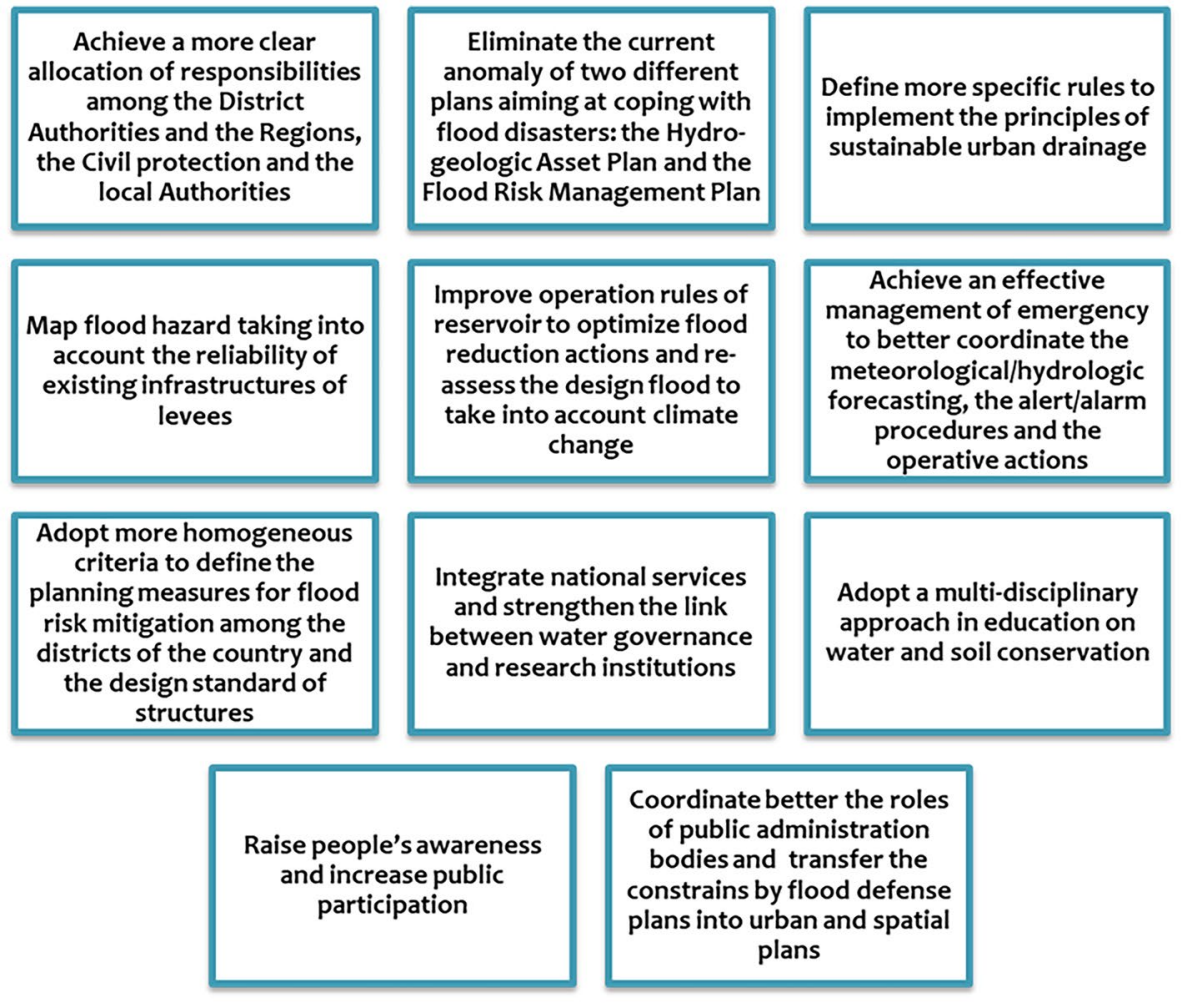
Priorities to increase the resilience of the Italian territory to flooding risk (adapted from Rossi and Benedini 2020)

The mentioned stages coincide substantially with the ones suggested by the Chart of the Sendai framework for disaster risk reduction, established by the United Nations Office for Disaster Risk Reduction (UNISDR 2015). The previous list proposed for the implementation of the European Flood Directive takes into account the recent directions of European policy concerning a high level of environmental protection in accordance with the principle of sustainable development and concerning the flexibility to be left to the local and regional authorities, according to the principles of proportionality and subsidiarity. Priorities to increase the resilience of the Italian territory to flooding risk are illustrated in Fig. 4, which include measures involving governance, policy, research, technical aspects, and citizens.

### Priorities for Coping with the Water-Food-Energy Nexus

The perspective that has led to adopt an integrated approach to water resources uses and water quality protection, today is extended by considering the need to face the future of water resources, energy production and food security within a comprehensive fashion, at the level of single countries and of the entire planet. As is well known, the main concerns regard the difficulty in satisfying the needs of agriculture (about 70% of total water needs) with decreasing water resources, not only due to pollution and climate change, but also to the increase in needs for other uses (civil, industrial and energetic). The risk of not being able to cover the agricultural production necessary to feed world population (expected to be almost 10 billion in 2050) is a serious issue, even taking into account the positive contribution of new low-water-demanding plant species and use of non-conventional resources (e.g., wastewater reuse). A review of the diet in developed countries is also invoked, for instance a reduction of meat consumption, which has a high water footprint. From an energetic point of view, the extent of the volumes of water used for energy production is not limited to the cooling of thermoelectric and nuclear power plants. For instance, currently there is also the need to meet the significant water needs for biofuel productions (in competition with agriculture for food production).

Adaptive water management is invoked to face specially the water-energy-food (WEF) nexus by linking the different strategies of development of each field and establishing efficient and effective tradeoffs and synergies between energy, water and food (Taguta et al. 2018).

Globalization of water resources has led to interconnections that frequently result in a complex interdependence between human civilizations and water, which implies links between human needs and local water resources to be treated with an integrated socio-environmental approach (Hoekstra and Chapagain 2008). The WEF nexus means that implemented solutions in one sector might primarily affect other sectors (Eftelioglu et al. 2017). The availability of water, for instance, affects how much food or energy can be produced. It follows that the challenges of managing water, energy, and food resources simultaneously, while achieving a number of potentially competing goals without harming the resource base of any sector, are significant and must be addressed as soon as practical (i.e., causing the least amount of damage to other sectors) (Olusola et al. 2022). Public policy and decision-making institutions, thus, play a key role in the WEF nexus. In this regard, different methods and regulations can be recognized in different nations. Italy's legal system, for instance, induces very strict quality limits for reuse of wastewater reuse in irrigation (Markantonis et al. 2019). This implies very high costs of wastewater treatment which are charged to the farmers. As a result, wastewater is released to the sea without any use, or wastewater is used illegally by farmers without any control.

With the above considerations, the first step toward creating an integrated assessment of WEF sectors is the formulation of sustainability indicators and a set of concrete criteria that weight resource management on a specific spatiotemporal scale (Hirwa et al. 2021). Moreover, approaches aimed at structuring discrete and interconnected linkages between water, human health, environment, and nutrition sectors by multi-criteria decision-making methods are under investigation (Nhamo et al. 2020; Rasul 2016). To move from theory to practice, it is necessary to pinpoint the economic and environmental sectors that are interconnected and could profit from a change in strategic planning. This crucial component appears to be not sufficiently investigated in the literature, but it would help in identifying areas with water supply issues, unequal resource allocations among various sectors of water users and in critical regions where food and energy demand is expected to increase over the coming decades. Among other goals, the outcomes could be an aid for long-term economic growth and environmental restoration (Olusola et al. 2022).

Adaptation measures to the issue of climate change and the WEF should be based on (Rasul and Sharma 2016): *i*) understanding the interdependence of subsystems within an overall system, focusing on system efficiency rather than the productivity of individual sectors; *ii*) recognition of interdependence between water, energy, and food and the promotion of economically rational decision making and efficient use of these resources in an environmentally responsible manner; *iii*) identification of integrated policy solutions to minimize trade-offs and maximize synergies across sectors; *iv*) attainment of policy coherence and coordination across sectors and stakeholders; and *v*) support of the transition to sustainability and consideration of the economic, social and ecological value of land, water, energy, and ecosystems.

## Concluding Remarks

The analysis made in the previous sections, making reference to the Italian case, has evidenced the main weaknesses of current water system management, which are however accompanied by some strengths. Weaknesses include: a sectorial approach to treat water issues; controversies among the different levels of governance, shortcomings in the maintenance of hydraulic facilities, delayed achievement of the objectives of water-related planning tools, prevalence of emergency actions on prevention measures for coping with the hydrological extremes, and a scarce preparation to the foreseeable impact of climate changes. On the other hand, strengths include: positive impacts on water quality and flood risk mitigation by European Directives, a growing role of national organisms improving the performance of sub-national bodies (for instance ARERA and Civil Protection), and positive impacts of recent unexpected world crisis of COVID-19. Regarding this last aspect, the fight against Coronavirus pandemic called great efforts for a more advanced asset of sanitary service, shifting financial resources towards the health needs and social help to low-income people. However, the resources of the European Union, through the national Recovery and Resilience Plan will allow to provide investments in water works for water supply and pollution control, as well as to adopt reforms in general policies. Also, the war in Ukraine and the impacts on gas supply to Europe, if on one side increase the risk of stopping the programs of reduction of emission of CO2, on the other side, reinforce the cooperation of European countries for a common energy’s policy and push to achieve solidarity practices to face the challenges of food security and improve the efforts for implementation of the “2030 Agenda for Sustainable Development Goals of the United Nations”.

The new paradigms to cope with climate and other global changes for water and soil management should be based on a comprehensive approach allowing to achieve sustainability in the triple consolidated dimension of economic, environmental and social dimension, as well as to improve the governance of water systems in terms of effective, equitable and ethical behavior. The proposed analysis of paradigm shifts in water resources exploitation and the attempt to identify strengths and weaknesses of current situation confirm that the increasing complexity of water resources management requires enlarged perspectives in the definition of an agenda for the future. In terms of water problems to face, an integrated water management, including issues of water supply, water quality and water-related risk, has to take into account also the links with food security and energy development.

Technical and management actions aimed at improving water services performance are key to face global changes. These actions include the adoption of advanced technologies applied to water quantity and quality monitoring, suitable pricing systems, robust ecological, economic and social indicators supporting decision making process. The capacity of facing the challenges of global changes requires also to consider the following priorities:

- an updated legislation to simplify the water-related plans and to achieve the objectives of the Climate change adaptation plan;
- better rules for regulating withdrawal from surface water bodies, that may include revising use authorizations in order to assure ecological flow and to avoid over-exploitation of groundwater;
- a reform of the responsible institutions, necessary for improving the coordination among the different bodies of the public administration, at central and regional levels;
- a better governance of municipal water services;
- transparent information to the water services customers, also for activating a responsible public participation;
- the implementation of the principle of hydraulic and hydrological invariance in the urban and land planning, in order to avoid the increase of peak flow and flood volume due to the growth of impervious areas;
- a better coordination in the warning and alert/alarm procedures of flood risk, particularly between municipalities and civil protection systems;
- a plan for mitigating the risk of drought and water shortage at district and water supply system levels.

Two conditions are preliminary to the specific measures to be adopted. The first is a multidisciplinary approach to face the water issue complexities. The second concerns the utmost importance of recognizing an ethical responsibility for water (Selborne 2000; Falkenmark and Folke 2002). Overall, the acceleration of the processes of global change requires a new “water culture” at all levels of responsibility, both in scientific and practical fields.

## Data Availability

N.A.
